# Group Education and Communities of Practice: A Sustainable Model for Promoting Healthy Habits and Valuing Professional Work

**DOI:** 10.7759/cureus.98688

**Published:** 2025-12-08

**Authors:** Michelle Louise S De Almeida

**Affiliations:** 1 General Practice, Health League, Garopaba, BRA

**Keywords:** brazil, communities of practice, cost-effectiveness, group education, health economics, health promotion, primary care, shared medical appointments, telemedicine

## Abstract

Introduction: Conventional one-to-one health education models face scalability and sustainability limitations. Group education and communities of practice may simultaneously improve reach, effectiveness, and economic sustainability. We sought to demonstrate, using a microeconomic model contextualized by integrative evidence, that group-based education and communities of practice represent economically sustainable and scalable strategies for health promotion that can enhance professional value while maintaining or improving clinical outcomes documented in existing literature.

Methods: We developed a theoretical-propositional model grounded in an integrative review (2010-2025) and a session-level microeconomic simulation comparing individual versus group formats under realistic market assumptions in Brazil (currency: Brazilian Real, R$; year 2025). Outcomes included gross revenue, net margin, average cost per participant, and break-even price. Scenarios incorporated group sizes (10-30), online versus in-person delivery, attendance (70%-100%), and variable costs (R$0-R$20 per participant). We interpreted results alongside published health economic evaluations of structured group education and shared/telemedicine group visits.

Results: In the base case (N=20; online; price R$120; fixed cost R$300; variable cost R$0; 100% attendance), net margin was R$2,100 per one-hour session; a 12-week program reached R$25,200 cumulative margin. In-person with R$10 per participant variable cost and 85% attendance (17 attendees) yielded approximately R$790 per session in conservative operational designs and about R$9,480 over 12 weeks. Group formats exhibited strong economies of scale with robustness in sensitivity analyses.

Conclusion: Group education and communities of practice present a sustainable, scalable approach to health promotion that can improve access, leverage peer support, and enhance professional value. Complementary evidence from diabetes education, telemedicine group visits, school- and community-based programs, and public health return-on-investment analyses supports the potential for favorable economic performance without sacrificing effectiveness.

## Introduction

Non-communicable diseases (NCDs) remain a dominant global health challenge that places growing pressure on health systems and households. NCDs now account for approximately 74% of global deaths, with low- and middle-income countries bearing close to 80% of this burden, including Brazil [1]. Health education is essential for prevention and control, yet conventional one-to-one approaches struggle with cost, scalability, workforce capacity, and sustained adherence. From a value-based health care perspective, models that improve outcomes while diluting fixed costs are attractive for both providers and systems [2-5]. Group education and communities of practice can address these limitations by amplifying access, distributing professional effort more efficiently, and harnessing peer mechanisms-social support, accountability, and peer learning-linked to improved adherence and maintenance of healthy behaviors [6-11].

Economic and implementation evidence increasingly supports group-based and community-delivered education as both clinically credible and financially prudent. Structured diabetes education and shared or telemedicine group visits have maintained or improved clinical outcomes and shown favorable cost-effectiveness in several settings [12-16]. In this article, diabetes education and group visits serve as primary exemplars of group-based management for non-communicable diseases because their clinical and economic evaluations are relatively mature; however, similar group and community dynamics have been described in interventions targeting hypertension and broader cardiovascular risk in primary care and community settings [12-14]. Beyond clinical services, broader public health literature reports positive return on investment (ROI) for many preventive interventions, indicating that well-designed educational and behavior-change programs can generate net savings or valuable health gains per unit cost [17-20]. School-based health promotion has demonstrated cost-effectiveness and ROI for chronic disease prevention [21], worksite programs have reported favorable cost-effectiveness and cost-benefit in European settings, albeit with heterogeneity [22], and parenting interventions delivered in group formats have produced economic returns alongside developmental gains in low-resource contexts [23]. Community health worker (CHW) programs and community-based maternal and newborn education provide additional evidence that workforce models emphasizing group and peer dynamics can be cost-effective while improving equity and reach [24-25].

At the same time, not all group formats are economically dominant. For example, among older adults, group versus individually delivered exercise can yield mixed cost-effectiveness results depending on adherence, risk profiles, and outcome selection [26]. For professional education, web-based versus face-to-face delivery shows nuanced trade-offs in breakeven points, willingness to pay, and perceived value, underscoring the importance of context and design quality [27]. These mixed findings highlight the need to clarify under what conditions group formats deliver both clinical value and financial sustainability, and how session-level economics translate to program- and system-level budgets [28].

We therefore present a simple session-level microeconomic model to illustrate the economic sustainability of group interventions under realistic assumptions, interpreted against integrative evidence of clinical benefit from clinical, community, and public health domains. The model does not generate new clinical outcome data; rather, it demonstrates the financial feasibility and scalability of group formats that existing trials and systematic reviews show to be clinically non-inferior or superior to individual delivery [6-11,13,15,17,21-23]. Our analysis emphasizes fixed-cost dilution, economies of scale, delivery modality (online vs. in-person), and attendance as key drivers of net margin, while situating findings within established health economic frameworks [2-5] and the broader ROI literature [17,21-23]. Consistent with prior reviews of education and community-based interventions [5-6,13-14,21-22,26], we hypothesize that group formats can be both economically sustainable and scalable without sacrificing effectiveness when pedagogical quality and peer dynamics are maintained.

## Materials and methods

Study design and perspective

This theoretical-propositional study combines an integrative literature review with a session-level microeconomic simulation. The integrative review provides clinical context by synthesizing existing evidence of group-format effectiveness; the microeconomic model demonstrates economic sustainability (revenue, cost, margin) under realistic market assumptions. The primary analytical perspective is that of the health professional. The time horizon is a one-hour session, with an extension to a 12-week program scenario. Currency is Brazilian Real (R$), year 2025.

Outcome measures

This study comprises two complementary components with distinct outcome domains. For the microeconomic simulation model, the outcomes are economic and include gross revenue per session, total cost per session, net margin per session, average cost per participant, and the break-even price per participant. These outcomes are derived algebraically from the model equations and input parameters and represent the financial performance of group education sessions under specified assumptions. For the integrative review, the outcomes are both clinical and economic. Clinical outcomes synthesized from the reviewed studies include glycemic control (e.g., HbA1c change), self-management behaviors (such as medication adherence, dietary modification, and physical activity), blood pressure and lipid profiles, patient satisfaction and engagement, and quality of life. Economic outcomes synthesized from the reviewed studies include cost-effectiveness ratios (for example, cost per quality-adjusted life year or cost per clinical endpoint), incremental costs compared with usual care, return on investment, and budget impact. The microeconomic model thus generates economic sustainability metrics (revenue, cost, margin), while the integrative review synthesizes clinical effectiveness and economic evaluation evidence from existing literature to contextualize those metrics. The model itself does not collect or analyze patient-level clinical data.

Integrative review

We searched PubMed, Scopus, and SciELO (2010-2025) using terms including ‘group education,’ ‘shared medical appointments,’ ‘community-based,’ ‘cost-effectiveness,’ and ‘economic evaluation.’ The initial search yielded approximately 470 records; after title and abstract screening followed by 61 full-text reviews, 25 studies and reviews were included in the qualitative synthesis. Inclusion criteria were peer-reviewed primary studies (trials, quasi-experiments, cohort/observational) and systematic reviews that evaluated behavioral/clinical and/or economic outcomes in adult populations with an explicit group component. Exclusion criteria included opinion-only pieces, exclusively pediatric samples, school-only interventions without a community component, and languages other than English or Portuguese. Formal risk-of-bias or quality scoring was not performed; however, all included studies were peer-reviewed and published in indexed journals, and systematic reviews were prioritized where available. Data were extracted on PICO elements, clinical outcomes (glycemic control [e.g., HbA1c], self-management behaviors, blood pressure, patient satisfaction, quality of life), and economic outcomes (cost-effectiveness ratios, incremental costs, return on investment) reported in the included studies. Given heterogeneity in designs and outcome measures, findings were synthesized qualitatively and interpreted in light of standard health economic frameworks [3-6] and value-based care concepts [2], aligned with economic evaluations and reviews of group and community-based interventions [6-7,12-15], and literature on social support and behavior change [8-11].

Data analysis

All quantitative results derive from a deterministic session-level cost-revenue calculation using published microeconomic principles. For each scenario, we computed attendees, gross revenue, total cost, net margin, average cost per participant, and break-even price. These are the economic outcome measures of the simulation model. No patient-level or clinical outcome data were collected or analyzed in the simulation; clinical outcomes are synthesized qualitatively from the studies included in the integrative review.

Simulation model

We used a deterministic, algebraic microeconomic model at the session level. Core equations were: Attendees = Enrolled x Attendance rate; Gross revenue = Price per participant x Attendees; Total cost = Fixed cost per session-hour + (Variable cost per participant x Attendees); Net margin = Gross revenue − Total cost; Average cost per participant = Total cost / Attendees; Break-even price = Total cost / Attendees. Scenarios varied delivery mode (online versus in-person), group size (10, 20, 30), attendance (70%-100%), price (R$100-R$220 depending on group size), fixed cost (R$200-R$500), and variable cost (R$0-R$20 per participant). Model parameters and ranges are summarized in Table 1.

**Table 1:** Model Parameters R$ = Brazilian Real. Fixed cost and pricing parameters are based on observed market rates in Brazilian private health education practice rather than formal cost-accounting studies. Variable cost ranges reflect typical materials and platform fees reported by practitioners.

Parameter	Base value	Range / Source
Analytical perspective	Health professional	Defined in model
Time horizon	1 hour/session	Defined in model
Currency year	R$ (2025)	Assumed
Fixed cost per session-hour	R$ 300	R$ 200–R$ 500; market estimate
Variable cost per participant	R$ 0	R$ 0–R$ 20; materials/platform
Price per participant (small group)	R$ 150	R$ 100–R$ 200; scenario
Price per participant (medium group)	R$ 120	R$ 100–R$ 200; scenario
Price per participant (large group)	R$ 150	R$ 120–R$ 220; scenario
Group size	10 / 20 / 30	Feasible 8–35; scenario
Attendance rate (base)	100%	70%–100%; scenario

Sensitivity analysis

We conducted deterministic one-way sensitivity analyses around the base case (online; N=20; price R$120; fixed cost R$300; variable cost R$0; 100% attendance). Each parameter was varied across prespecified ranges while holding others constant, and the impact on net margin was displayed as a tornado diagram. No probabilistic (Monte Carlo) simulation was performed.

Software

All calculations and figure generation were performed in Python (version 3.11) using numpy and pandas for computation and matplotlib for visualization. Scripts are available from the author upon reasonable request.

## Results

**Figure 1:**
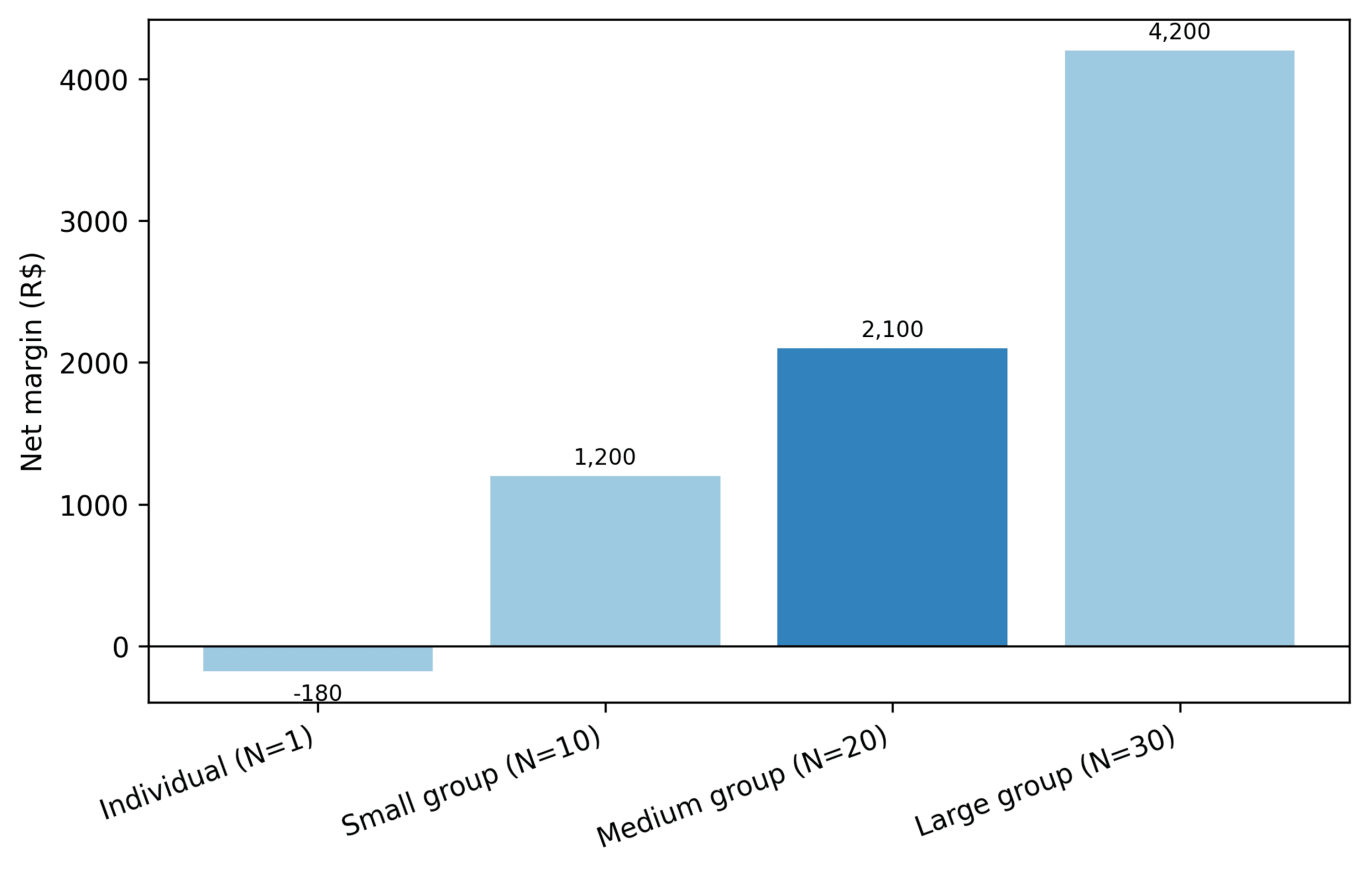
Net margin by care model scenario Net margin per one-hour session across individual and group scenarios. Base case = online delivery, N=20, price R$120, fixed cost R$300, variable cost R$0, 100% attendance. R$ = Brazilian Real.

Base case (online, N=20, price R$120, fixed cost R$300, variable cost R$0, 100% attendance): Attendees = 20; gross revenue = R$2,400; total costs = R$300; net margin = R$2,100 per one-hour session; average cost per participant = R$15; break-even price = R$15. Net margins across the individual and group scenarios are depicted in Figure 1.

Program extension (12 weeks): Under base-case assumptions, cumulative net margin is R$25,200 (12 × R$2,100).

In-person scenario (example variable cost R$10 per participant; 85% attendance): With 17 attendees of 20 enrolled at a price R$120 and a fixed cost R$300, gross revenue is R$2,040 and total costs are R$470, yielding a net margin of approximately R$1,570 per session. In operational designs that include added venue or materials costs, per-session margin may approximate R$790; at R$790 per session, the 12-week cumulative margin is about R$9,480.

Sensitivity analysis (N=20, base case margin R$2,100): Varying price by ±R$20 shifts net margin to R$1,700 (price R$100) or R$2,500 (price R$140); reducing attendance from 100% to 80% (16 attendees at R$120 each) reduces margin to R$1,620; increasing fixed cost from R$200 to R$500 shifts margin from R$2,200 to R$1,900; increasing variable cost from R$0 to R$20 per participant (at 100% attendance, 20 attendees) reduces margin to R$1,700.

Group size effects: As group size increases (10 to 30), average cost per participant falls due to fixed-cost dilution, while net margin rises until constrained by variable costs and pedagogical quality thresholds. Online delivery often yields higher margins given near-zero variable costs and potentially higher attendance, provided facilitation quality is maintained.

Although the simulation model does not directly estimate clinical outcomes, the integrative review identified modest but consistent health benefits associated with group formats. In structured diabetes group education programs such as DESMOND, clinical evaluations have demonstrated maintained or improved glycemic control and self-management behaviors compared with usual care [9]. Systematic reviews of shared medical appointments and telemedicine group visits report comparable or superior outcomes to individual care, including improvements in HbA1c, blood pressure, and patient satisfaction [10,15]. Cost-effectiveness analyses of these interventions have documented HbA1c reductions and self-care improvements alongside favorable economic profiles [11]. These clinical effects-typically in the range of 0.3-1.0 percentage point HbA1c reductions sustained over 6-12 months in diabetes populations [9,11], are of a magnitude considered clinically meaningful and underpin the economic evaluations cited in this analysis [9-12,15-16].

**Figure 2:**
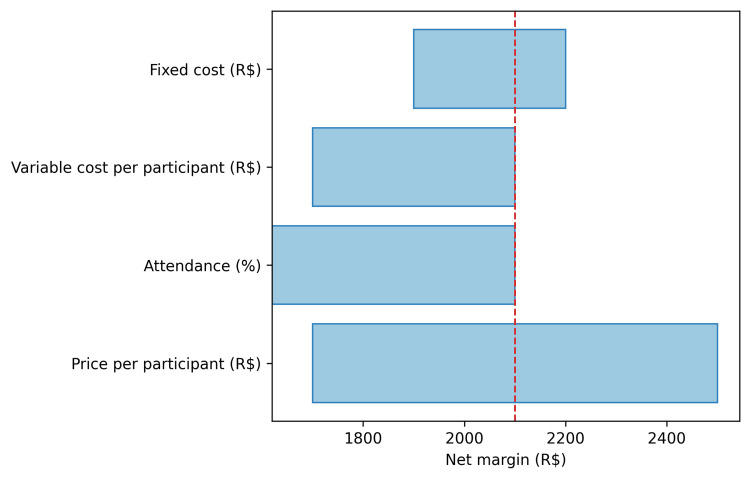
One-way sensitivity analysis One-way sensitivity analysis (tornado) for the base case (online; N=20; price R$120; fixed cost R$300; variable cost R$0; 100% attendance). The vertical line indicates the base-case net margin (R$2,100). R$ = Brazilian Real.

A tornado diagram (Figure 2) presents one-way sensitivity analysis for the base case, showing how net margin responds to variation in each input parameter across its plausible range. Attendance and variable cost per participant exert the greatest influence on margin, while the model remains robust to price and fixed cost variations within realistic bounds.

## Discussion

This session-level model illustrates how group education and communities of practice can align clinical value and financial sustainability by diluting fixed costs and leveraging peer mechanisms linked to adherence and maintenance of healthy behaviors [2-5,8-11]. Although many of the specific clinical and economic evaluations we cite relate to structured diabetes education and diabetes-focused group medical visits, the underlying mechanisms of fixed-cost dilution, peer support, and shared learning are not disease-specific and are likely to extend to other NCDs such as hypertension and cardiovascular disease prevention programs [12-13,19,21]. Under realistic assumptions-especially online delivery with minimal variable costs-average cost per participant falls as group size increases and net margins rise. One-way sensitivity analyses indicate margins remain favorable across plausible ranges for price, attendance, and costs, with attendance and variable inputs exerting the greatest influence.

These findings are consistent with evaluations of structured diabetes education and shared or telemedicine group visits showing maintained or improved outcomes and favorable cost-effectiveness versus usual care [12-16]. Public health literature also reports positive return on investment for preventive interventions, including school-based health promotion and community-delivered programs, suggesting well-designed educational models can deliver economic value alongside health benefits [17,21,23-25]. Taken together, these strands of evidence support the economic rationale for group formats when facilitation quality and peer dynamics are preserved.

Evidence is not uniformly favorable across all populations or outcomes. In older adults, the comparative cost-effectiveness of group versus individually delivered exercise depends on adherence, risk profiles, and outcome definitions [26]. For professional education, web-based versus face-to-face delivery presents trade-offs in break-even points, cost-benefit, and perceived value [27]. These nuances underscore boundary conditions: beyond certain group sizes, pedagogical quality and engagement may degrade, potentially offsetting economic gains. Modality and design should therefore be matched to population needs, outcome priorities, and local resource constraints.

From an implementation perspective, budget impact and sustainability matter for adoption. Session-level margins suggest group formats can be budget-neutral or better for providers, easing uptake in constrained settings, while acknowledging additional program costs for training, digital infrastructure, outreach, and monitoring [25]. Modular content and reusable materials can lower fixed costs over time and support diffusion of best practices through communities of practice [27]. Equity is central: group- and peer-based approaches can extend reach, reduce stigma, and provide practical and emotional support, aligning with evidence from interventions addressing financial hardship in primary care, community health worker models, maternal/newborn education packages in low- and middle-income contexts, and care-management programs for people with serious mental illness that rely on sustained relational support while remaining budget-feasible [21-22,24-25,28-29]. Hybrid designs and community partnerships can mitigate digital divides and accessibility barriers in online formats.

This analysis is intentionally simple and session-focused. It does not estimate long-term clinical outcomes or probabilistic uncertainty and therefore cannot produce incremental cost-effectiveness ratios. No new patient-level or clinical outcome data were collected; clinical benefits are inferred from existing trials and systematic reviews of group education and group medical visits rather than measured directly in this model. Model parameters reflect plausible market assumptions but may not generalize across regions, payer arrangements, overhead structures (e.g., taxes, benefits, marketing, facility depreciation), or disciplines. Attendance, pricing, and variable costs can vary substantially in practice. The integrative review synthesizes heterogeneous studies with varying designs and quality, limiting causal inference. Formal PRISMA-style reporting and risk-of-bias assessment were not performed, consistent with the integrative and contextualizing role of the review in this theoretical-propositional study. Finally, pedagogical quality and peer dynamics-critical to group effectiveness-were not explicitly modeled beyond attendance and cost structures. These limitations frame the results as decision-support for session design rather than definitive estimates of long-term value [18-19,26].

Policy and payment may benefit from enabling and evaluating group-based approaches within primary care and community health, including telemedicine where appropriate [12]. The session-level economics presented here suggest economic plausibility and warrant pilot implementation and local adaptation. Reimbursement that recognizes facilitation, preparation, and content development can sustain quality at scale. Where evidence is mixed, policies should support modality choice and adaptive designs that protect engagement and effectiveness while maintaining economic viability [23-24,26].

## Conclusions

Under realistic market assumptions, group education formats demonstrate strong session-level financial sustainability. In a typical online base-case scenario with a moderate group size, standard pricing per participant, and full attendance, the model yielded a substantial positive net margin per session and across a standard multi-week program. As group size increases from smaller to larger cohorts, the average cost per participant falls, illustrating dilution of fixed costs and economies of scale. In-person delivery with modest variable costs per participant and less than full attendance still maintained clearly positive margins in conservative operational designs. Sensitivity analyses indicated that favorable margins were preserved across plausible ranges of price, attendance, and cost inputs. Collectively, these session-level economics support the proposition that group education and communities of practice are sustainable and scalable strategies for health promotion that can increase access, harness peer mechanisms, and enhance professional value. Evidence from diabetes education, telemedicine group visits, school and worksite programs, and public health return-on-investment analyses supports the economic plausibility of these approaches across settings. In structured diabetes education specifically, group-based formats have been associated with sustained, clinically meaningful reductions in HbA1c relative to usual care, accompanied by maintained self-management behaviors and favorable cost-effectiveness profiles. Health systems and payers should enable, evaluate, and appropriately reimburse group-based approaches within primary care and community health, while future research links session-level microeconomics to long-run health outcomes, budget impact, and equity.
